# Pros & cons: impacts of social media on mental health

**DOI:** 10.1186/s40359-023-01243-x

**Published:** 2023-07-06

**Authors:** Ágnes Zsila, Marc Eric S. Reyes

**Affiliations:** 1 Institute of Psychology, Pázmány Péter Catholic University, Budapest, Hungary; 2 Institute of Psychology, ELTE Eötvös Loránd University, Budapest, Hungary; 3 Department of Psychology, College of Science, University of Santo Tomas, Manila, 1008 Philippines

**Keywords:** Social media, Mental health, Impact, Risks, Benefits

## Abstract

The use of social media significantly impacts mental health. It can enhance connection, increase self-esteem, and improve a sense of belonging. But it can also lead to tremendous stress, pressure to compare oneself to others, and increased sadness and isolation. Mindful use is essential to social media consumption.

Social media has become integral to our daily routines: we interact with family members and friends, accept invitations to public events, and join online communities to meet people who share similar preferences using these platforms. Social media has opened a new avenue for social experiences since the early 2000s, extending the possibilities for communication. According to recent research [1], people spend 2.3 h daily on social media. YouTube, TikTok, Instagram, and Snapchat have become increasingly popular among youth in 2022, and one-third think they spend too much time on these platforms [2]. The considerable time people spend on social media worldwide has directed researchers’ attention toward the potential benefits and risks. Research shows excessive use is mainly associated with lower psychological well-being [3]. However, findings also suggest that the quality rather than the quantity of social media use can determine whether the experience will enhance or deteriorate the user’s mental health [4]. In this collection, we will explore the impact of social media use on mental health by providing comprehensive research perspectives on positive and negative effects.

Social media can provide opportunities to enhance the mental health of users by facilitating social connections and peer support [5]. Indeed, online communities can provide a space for discussions regarding health conditions, adverse life events, or everyday challenges, which may decrease the sense of stigmatization and increase belongingness and perceived emotional support. Mutual friendships, rewarding social interactions, and humor on social media also reduced stress during the COVID-19 pandemic [4].

On the other hand, several studies have pointed out the potentially detrimental effects of social media use on mental health. Concerns have been raised that social media may lead to body image dissatisfaction [6], increase the risk of addiction and cyberbullying involvement [5], contribute to phubbing behaviors [7], and negatively affects mood [8]. Excessive use has increased loneliness, fear of missing out, and decreased subjective well-being and life satisfaction [8]. Users at risk of social media addiction often report depressive symptoms and lower self-esteem [9].

Overall, findings regarding the impact of social media on mental health pointed out some essential resources for psychological well-being through rewarding online social interactions. However, there is a need to raise awareness about the possible risks associated with excessive use, which can negatively affect mental health and everyday functioning [9]. There is neither a negative nor positive consensus regarding the effects of social media on people. However, by teaching people social media literacy, we can maximize their chances of having balanced, safe, and meaningful experiences on these platforms [10].

We encourage researchers to submit their research articles and contribute to a more differentiated overview of the impact of social media on mental health. *BMC Psychology* welcomes submissions to its new collection, which promises to present the latest findings in the emerging field of social media research. We seek research papers using qualitative and quantitative methods, focusing on social media users’ positive and negative aspects. We believe this collection will provide a more comprehensive picture of social media’s positive and negative effects on users’ mental health.

## Data Availability

Not applicable.
